# Improving adherence to ‘test, trace and isolate’

**DOI:** 10.1177/0141076820956824

**Published:** 2020-09-10

**Authors:** G James Rubin, Louise E Smith, GJ Melendez-Torres, Lucy Yardley

**Affiliations:** 1 Weston Education Centre, King’s College London, London SE5 9RJ, UK; 2 College of Medicine and Health, University of Exeter, Exeter EX1 2LU, UK; 3 School of Psychological Science, University of Bristol, Bristol BS8 1TU, UK; 4 Department of Psychology, University of Southampton, Southampton SO17 1BJ, UK

Isolating sick patients, tracing their contacts and placing them into quarantine is a strategy used by generations of public health physicians in their fight against infectious disease. As coronavirus lockdowns around the world start to ease, new systems based on this strategy are being rolled out to prevent a resurgence of the pandemic. If these systems do not consider the behavioural issues posed by symptom reporting and quarantine, they will struggle to meet their goals.

The goals of this commentary are to outline the key behavioural issues that a test, trace and isolate system for the management of COVID-19 will face, and to suggest specific ways that system design can address these. In this paper, we highlight five areas in which these behavioural issues should be taken into account, structured to follow the steps of the test, trace and isolate process: development of symptoms; reporting of symptoms; isolation; reporting of contacts; and quarantining contacts. The issues we highlight largely apply regardless of the modality of system, be it via an app, website or in-person consultation.

## Development of symptoms

The initial trigger for any test, trace and isolate system begins with a member of the public developing symptoms. Unfortunately, the main symptoms of COVID-19 (new continuous cough or a high temperature) are non-specific: most people with these symptoms will not have COVID-19 (see Box 1).

**Box 1.** A cross-sectional survey of a representative sample of UK adults.

• A cross-sectional survey of the UK adult population (18 years or older) was commissioned by the market research organisation YouGov. Full details of the method are available elsewhere.^1^
• 2240 respondents were recruited from YouGov’s online panel.
• Quota sampling was used, with quotas based on age, gender and Government Office Region.
• Fieldwork was undertaken 6th – 7th May.
• Data are reported unweighted.
• Relevant questions were:
○ In the past seven days, which, if any, of the following symptoms have you experienced? (Response options included cough and high temperature/fever).
○ In the past 14 days, which, if any, of the following symptoms has someone else in your household experienced? (Response options included cough and high temperature/fever).
○ In the past 24 hours, how many times, if at all, have you left your home for each of the following reasons? (Seven reasons, such as ‘for exercise’, were listed).
• We calculated the proportion of participants who reported having either cough or fever themselves as 6.5% (n = 146). The best estimate of COVID-19 prevalence in England for the same period is 0.25%.^2^
• We used a chi-squared test to look for an association between the presence of symptoms in either the respondent themselves or a household member (symptoms present n = 217, symptoms not present n = 1945), and whether the respondent had left their home at least once in the past 24 hours. This showed no significant association (χ^2^(1, 2147) = 0.54, *p* = 0.46).

Over the course of any major public health incident, symptoms that arise or are exacerbated as a result of heightened anxiety or media reporting are also likely. In other major incidents, the number of patients reporting possible exposure to a hazardous substance or infection and who are eventually found to be unaffected has dwarfed the number of genuine cases by as much as 100 to 1. Even if only 1% or 2% of the UK population experience new continuous dry cough or fever, that will still represent an enormous workload for any test, trace and isolate system to cope with. Many patients testing negative will have their own, varied health needs and will require targeted reassurance, guidance and, occasionally, onward referral to other health services.^3^ Patients suffering from health anxiety may prove difficult to reassure and may take up a disproportionate amount of staff time.

## Reporting of symptoms

Patients with cough and fever will only make contact with services if they recognise that these may be indicators of COVID-19. Media reports have spotlighted newsworthy but peripheral symptoms (e.g. ‘COVID toe’, delirium). Surveys of the UK population suggest only 59% identify cough, fever and anosmia as ‘red flag’ symptoms.^4^ Basic factual education regarding the symptoms of COVID-19 will be a pre-requisite for the success of any service.

Even where patients recognised relevant symptoms, the range of other, more likely explanations will likely delay or dissuade reporting,^5^ especially for mild symptoms. During the early stages of the H1N1v pandemic, willingness to report symptoms was linked to severity.^3^ Media campaigns urging patients not to self-diagnose and not to ‘wait and see’ whether mild symptoms resolve by themselves will be needed to encourage speedy reporting, particularly as waiting to see if mild symptoms resolve before reporting them is a frequent message to patients in other contexts.

Other factors are also likely to stand in the way of timely reporting. For example, worry is associated with symptom perception and use of medical services,^6^ but worry about COVID-19 is showing a slow but steady decline in the UK.^7^ Concern about using a service properly, being perceived as a ‘time waster’, and practical aspects such as ease of access and competing demands on your time will also put people off reporting their symptoms.^5^ Conversely, pressure from friends, family or employers to ‘get checked out’ may provide motivation to report,^3^ as will the offer of a test to resolve uncertainty.

## Isolation

Isolation is unpleasant and distressing^8^ and can lead to severe financial and social costs. It results in no immediate benefit for the index case and is largely an act of altruism. Unless people are convinced that they will be fully and quickly recompensed for any financial cost and that their use of the test, trace and isolate system is both expected and respected by their community, then, particularly where symptoms are mild, it may be tempting for some to accept their first assumption that their symptoms are probably unrelated to COVID-19. Reducing the costs associated with use of the service will be essential to improving its uptake; this could include an early release from isolation if a negative test result is obtained. Prioritising rapid testing, especially as some households may develop symptoms multiple times, is therefore essential to the success of the service.

Among those who test positive, the challenges of maintaining isolation for the full duration, even among those motivated to try, should not be ignored.^9^ We used our polling to compare people who reported cough or fever in their household with those who did not. There was no difference in the total number of times people in each group had left their homes in the past 24 hours. Only 25% of those in the symptomatic group had followed government guidelines to not leave their home at all. Identifying ways to promote adherence to isolation must therefore be a priority. In our polling, receiving help from others from outside the home was linked to people being less likely to break isolation. Ensuring that a test, trace and isolate system links people up with community support mechanisms may help promote adherence.

When the period of isolation is over, people who have tested positive will need to be warned that we still do not know if people can develop COVID-19 more than once and that they must still be careful to avoid spreading infection. In the absence of guidance, social distancing can decrease in those who believe (rightly or wrongly) they have had COVID-19.^10^

## Reporting of contacts

Contact tracing only works if the index case is both able and willing to identify their contacts. App-based approaches, which record contacts between app-users automatically and facilitate alerting, are one solution, but come with their own drawbacks^11^ and are limited by uptake rates. More traditional methods of contact tracing require the index case to recall information about who they have been close to, know their contact details and volunteer that information. Willingness to volunteer this information is not necessarily straightforward. People may be disinclined to cause disruption for some of their contacts, particularly if the cause of that disruption can be linked back to them. For example, one voluntary group’s attempt at a small COVID-19 contact-tracing service in Sheffield reported that employers dissuaded some of their staff from reporting contacts from within the organisation.^12^ Guaranteeing that people may use the service confidentially, and that their details will be well-guarded against accidental disclosure, is essential if people are to trust the system.^11^ Giving people the option to notify contacts themselves rather than having someone else, or an app, undertake this task may increase cooperation and decrease the perception of intrusiveness. Relatedly, some evidence from contact tracing for sexually transmitted infections via geosocial networking apps suggests automatic notification may be preferred for casual partners, whereas contact notification for proximal relationships is still preferred.^13^

## Quarantining contacts

Alerting contacts that they may have been in touch with an index case can occur in a variety of ways, including a message on a mobile phone app, an SMS, an email or a phone call. Bogus contract tracing messages are already known to be circulating, which will reduce engagement with genuine messages. An electronic alerting system will therefore require a mechanism for people to verify a message. Contact tracing is also viewed as more acceptable against a background of high levels of trust in an individualised provider. Personalising that provider as, for example, a health visitor or other public health clinician can increase acceptability of contact tracing, even where contact tracing is undertaken using a technological solution.

The anonymity of the test, trace and isolate system and quarantine’s financial and social impact may also work against the likelihood of a contact’s compliance. If employers or neighbours are unaware that the individual is meant to be in quarantine, there will be limited social support or pressure to remain at home. Altruism will therefore be a key motivator. Contact tracing is viewed as more acceptable when framed in terms of an appeal to duty and being ‘the right thing to do’.^14^

The large volume of symptoms in the community mean that some people, particularly those with many contacts, might be asked to quarantine multiple times. Some proposed systems therefore only isolate contacts based on a positive test result in the index case. However, for some people, particularly those with regular contact with people who are clinically vulnerable, a very early warning that they may be at risk may be valuable. This may allow them to avoid or reduce contact with vulnerable people while the index case awaits results.

## Conclusion

Implementing the test, trace and isolate system poses a complex set of behavioural challenges for the individual, involving self-diagnosis, self-referral and self-management. As with other contexts,^15^ adherence to these steps will be improved if we ensure that people understand exactly when and how to act, are motivated by perceived personal and sociocultural benefits, and are helped to overcome the many practical and social barriers to adherence.

## Declarations

### Guarantor

GJR.
